# Before attributing improved sleep quality to an anti-inflammatory diet, alternative causes must be ruled out

**DOI:** 10.1590/1806-9282.20241580

**Published:** 2025-06-02

**Authors:** Josef Finsterer

**Affiliations:** 1 Neurology and Neurophysiology Center, Department of Neurology – Vienna, Austria.

Dear Editor,

We were interested to read the article by Toğuç et al. on a controlled single-blind study on the influence of an anti-inflammatory diet (AID) on inflammatory biomarkers, anthropometric indices, and sleep quality in 16 obese patients who received an AID diet and 17 patients who received a control diet (CD) for 8 weeks^1^. It was found that both AID and CD lowered body mass index (BMI) and C-reactive protein (CRP) in both groups but did not significantly improve sleep quality^1^. It was concluded that AID can improve BMI, inflammation, and sleep in obese patients^1^. The study is impressive, but some points should be discussed.

The first point is that factors other than obesity and diet that influence inflammatory parameters, anthropometric indices, and sleep quality were not sufficiently taken into account. Inflammatory parameters can also be strongly influenced by the presence or absence of subclinical chronic or acute infectious diseases (e.g., dental foci), immunologic diseases, and occult malignancies. The quality of sleep can be highly dependent on endogenous and exogenous factors, such as acute or chronic stress, the balance between sympathetic and parasympathetic tones, hormonal balance, medication, drugs, alcohol, nicotine, cola, Red Bull, environmental factors (noise, humidity, and temperature), nutritional rhythms, the amount of food, the amount and timing of fluid intake, and exposure to electrosmog. Therefore, we should know how these conditions could be excluded as a cause of increased inflammatory parameters or sleep disturbances.

The second point is that it was not reported how compliance with AID was controlled. Was AID adherence monitored at all and by what means? Was blood taken regularly from the patients, and, if so, which parameters were examined?

The third point is that the study was conducted during the pandemic between November 2021 and May 2022^1^. Therefore, we should know how many of the included patients were SARS-CoV-2 positive during the study and to what extent COVID-19 affected their dietary habits.

The fourth point is that obesity can also be caused by psychiatric disorders. Depression and anxiety in particular can have a strong influence on appetite. Therefore, it would have been mandatory to also assess how many of the included patients were obese due to depression, using one of the common depression scales. In this context, we should also know the current medication of each included person, as medication can strongly influence eating habits.

The fifth point is that it is not reported what kind of inflammatory markers besides CRP were actually determined. Was only CRP determined in the patients or were ferritin, D-dimer, procalcitonin, interleukin-6, or alpha-2-globulin also determined? It also remains unclear whether the reduction in BMI or the reduction in CRP was responsible for the improved sleep quality.

In summary, it can be said that the index study has limitations that relativize the results and their interpretation. Addressing these limitations could strengthen the conclusions and support the results of the study. Before attributing the improved sleep quality in obese patients to AID, other causes of sleep disturbance must be ruled out.
